# Body fat indicators for cardiometabolic risk screening among shift workers

**DOI:** 10.47626/1679-4435-2020-440

**Published:** 2020-12-11

**Authors:** Amanda Popolino Diniz, Márcia Elivane Alves, Virgínia Capistrano Fajardo, Silvia Nascimento de Freitas, Guilherme Augusto Sousa Batista, Bruno Francia Maia Athadeu, George Luiz Lins Machado-Coelho, Fernando Luiz Pereira de Oliveira, Fausto Aloísio Pedrosa Pimenta, Raimundo Marques do Nascimento Neto

**Affiliations:** 1 Pós-Graduação em Saúde e Nutrição, Universidade Federal de Ouro Preto - Ouro Preto (MG), Brazil; 2 Pós-Graduação em Ciências Aplicadas à Saúde do Adulto, Universidade Federal de Minas Gerais - Belo Horizote (MG), Brazil; 3 Escola de Medicina, Universidade Federal de Ouro Preto - Ouro Preto (MG), Brasil; 4 Departamento de Medicina de Família, Saúde Mental e Coletiva, Universidade Federal de Ouro Preto - Ouro Preto (MG), Brazil; 5 Departamento de Estatística, Universidade Federal de Ouro Preto - Ouro Preto (MG), Brazil; 6 Departamento de Clínicas Pediátrica e do Adulto, Universidade Federal de Ouro Preto - Ouro Preto (MG), Brazil

**Keywords:** obesity, circadian rhythm, body weight, anthropometry

## Abstract

**Background:**

In view of the costly methods currently available for the assessment of body adiposity, anthropometric obesity indicators have proven effective in predicting cardiovascular risk.

**Objective:**

To investigate the discriminatory power of body fat indicators for cardiovascular risk screening among shift workers.

**Methods:**

Cross-sectional study with male employees of an iron ore extraction company. The predictive power of body fat indicators relative to cardiovascular risk was analyzed based on the Framingham risk score and metabolic syndrome by means of receiver operating characteristic curves, sensitivity, specificity, positive and negative predictive values, area under the receiver operating characteristic curve and Youden’s index.

**Results:**

The prevalence of cardiovascular risk was 14.2% in the metabolic syndrome risk model. According to the Framingham score, 95.0%, 4.1% and 0.9% of the participants exhibited low, moderate and high risk, respectively. All the analyzed body fat indicators exhibited satisfactory discriminatory power for the tested cardiovascular risk models.

**Conclusion:**

Waist-height ratio exhibited the highest ability to predict cardiometabolic risk in both risk models.

## INTRODUCTION

Obesity, understood as excess body fat, is a multifactorial disease that causes inflammation, which eventually leads to metabolic disorders.^1^ Obesity has a direct relationship with cardiovascular (CV) disease and risk factors, including dyslipidemia, high blood pressure (BP), insulin resistance and diabetes.^1^ While several methods are available to measure body fat, as a rule they involve expensive and sophisticated equipment, which hinders their applicability and accessibility in clinical practice and epidemiological studies.^1^ Screening for CV risk requires less expensive methods. Anthropometric indicators, such as neck circumference (NC), waist circumference (WC), body mass index (BMI) and waist-to-height ratio (WHtR), have proven helpful in this regard, but there is no consensus yet about the best predictor of CV risk.^1^^-^^6^

CV disease is the leading cause of death worldwide. In 2012 it accounted for 17.7 million deaths and 46% of all mortality due to noncommunicable diseases.^7^ The main behavioral risk factors for CV disease are smoking, physical inactivity, unhealthy eating and alcohol abuse. Shift-related changes in circadian rhythm seem to be an additional risk factor.^8^ Shift work is understood as any daily work schedule outside of standard working hours, such as night work and rotating rosters.^9^ This type of schedule interferes with endogenous circadian rhythm and may cause endocrine and metabolic disorders, including abnormal BMI, triglyceride, high-density lipoprotein (HDL) and blood sugar levels.^10^ Shift workers are more prone to obesity due to changes in dietary habits, sedentary lifestyle and circadian rhythm disruption, which suggests they are at higher risk of CV disease.^9^

Although risk factors independently impact CV outcomes, they are frequently combined in the same individual. This factor is considered in risk models for the prevention and early detection of CV disease, such as the Framingham risk score (FRS) and metabolic syndrome (MS).^11^ The former is a simplified tool to estimate 10-year CV risk according to risk factors for coronary artery disease (age, sex, total cholesterol, HDL, smoking and systolic BP).^12^

The latter is a set of metabolic CV risk factors that includes insulin resistance, central obesity, diabetes and hyperlipidemia.^13^ According to several studies, the simultaneous presence of several risk factors increases the odds of CV events.^14^^,^^15^ Therefore, studies involving risk models such as FRS and MS are relevant as a basis for preventive actions and interventions.

The aim of the present study was to establish the discriminatory power of body fat indicators in CV risk screening among workers with rotating shifts.

## METHODS

The present cross-sectional study included male employees of an iron mining company near Inconfidentes, Minas Gerais, Brazil. The employees worked 36 hours per week as follows: a 6-hour shift (19:00 to 01:00, 01:00 to 07:00, 7:00 to 13:00, or 13:00 to 19:00) followed by a 12-hour rest, as well as one full day off work after each four-shift cycle.

Data collection was performed at outpatient clinics on the company premises by investigators previously trained to administer the questionnaires and perform anthropometric and BP measurements. The participants also responded to a structured sociodemographic questionnaire to investigate sex, educational level, ethnicity and marital status. The results are shown in Table 1.

**Table 1:** Prevalence of sociodemographic characteristics of rotating shift workers. Inconfidentes, Brazil, 2011 (n = 678)

Variables/categories	n	%
Age group		
20-39 years	169	24.9
30-40 years	336	49.6
>40 years	173	25.5
Marital situation		
Married	456	67.3
Not married	221	32.7
Race		
White	220	35.4
Non-white	438	64.6
Education level		
Incomplete elementary school	16	2.4
Complete elementary school	58	8.6
Complete high school	589	86.9
Complete higher education	15	2.2
Framingham risk score		
Low	644	95.0
Average	28	4.1
High	6	0.9
Metabolic syndrome (NCEP-ATP III)/present	96	14.2
Blood pressure/SBP ≥ 130 mmHg, DBP ≥ 85 mmHg	343	50.6
HDL/<40mg/dL	162	23.9
Triglycerides/≥150mg/dL	240	35.4
NCEP-ATP III/≥110mg/dL	17	2.5
Waist circumference (NCEP-ATP III)/>102 cm	104	15.3

DBP: diastolic blood pressure; HDL: high density lipoprotein; NCEP-ATP III: Na- tional Cholesterol Education Program Adult Treatment Panel III; SBP: systolic blood pressure.

Body weight was measured with Tanita^(®)^ BC-554 portable scale (Biospace Co. Ltd. Factory, Korea)^16^ and height was measured with an Alturexata^(®)^ stadiometer (Belo Horizonte, Brazil).^16^ These measurements were used to calculate BMI (weight/height^2^).^17^ Obesity was defined according to World Health Organization parameters (BMI ≥ 25 kg/m^2^). NC and WC were measured three times with a non-elastic tape measure. WC was measured at the midpoint between the iliac crest and the last rib.^16^ Participants with a WC ≥ 102 cm were considered to have central obesity.^18^ WHtR was calculated by dividing NC (cm) by height (cm)^19^. High WHtR was defined as ≥0.50.^19^ NC was measured at the level of the cricothyroid cartilage right above the laryngeal prominence^20^ and was rated as high when ≥39.5 cm.^21^

BP was measured using a HEM-705CP digital monitor (Omron Healthcare, Inc., IntelliSense, Bannockburn, Illinois, USA) and an appropriate cuff size was used for each arm circumference. The measurements were performed with the participants sitting and their right arm at the level of the heart.^22^ High CV risk was defined as systolic BP > 130 mmHg and/or diastolic BP > 85 mmHg.^23^

Participants were required to schedule blood collection appointments at the company’s medical department. The enzymatic-colorimetric method was used to measure 12-hour fasting blood glucose, triglycerides, total cholesterol and fractions. Sample collection, processing and analysis were performed at a laboratory hired by the employer.

### FRAMINGHAM RISK SCORE AND METABOLIC SYNDROME

FRS was calculated based on age, systolic and diastolic BP, total cholesterol and HDL levels, smoking and diabetes.^23^ Ten-year CV risk was categorized as low when ≤10%, moderate when 10-19% or high when ≥20%.^23^^,^^24^ For the purposes of analysis, we considered low and moderate/high risk.

MS was analyzed according to the National Cholesterol Education Program Adult Treatment Panel III criteria.^18^ MS was diagnosed when at least three of the following criteria were found: abdominal obesity (WC ≥102 cm for males), elevated triglyceride level (≥150 mg/dL), low HDL (men: <40 mg/dL), high BP (systolic ≥ 130 or diastolic ≥ 85 mmHg) and high fasting blood sugar (≥110 mg/dL).

### STATISTICAL ANALYSIS

Descriptive analysis included absolute and relative frequencies for categorical variables and mean (± standard deviation), median, minimum and maximum values for continuous variables. Normality was investigated with the Kolmogorov-Smirnov test. We plotted receiver operating characteristic (ROC) curves to analyze the predictive ability of body fat indicators for CV risk. We calculated the area under the curve (AUC) for anthropometric indicators (WHtR, WC, BMI, NC) and risk models (FRS and MS). The significance level was set at 5%. The AUC of the anthropometric variables were compared with the Hanley and McNeil method. The cut-off points for body fat indicators were based on the highest Youden’s indices with significant AUC and corresponding sensitivity, specificity, positive and negative predictive value. Descriptive analysis, normality testing and sample size calculation were performed in SPSS version 22.0 and OpenEpi version 3.01. MedCalc version 18.2.1 was used to plot the ROC curves and the Hanley and McNeil test.

The study was approved by the research ethics committee of Universidade Federal de Ouro Preto (CAAE: 0018.0.238.000-11, ruling no. 074/2011). All participants provided written informed consent.

## RESULTS

Of 952 eligible subjects, 274 refused to participate or were on vacation or sick leave. Although the final sample included 678 participants, it was representative in terms of work schedule (Appendix 1, available as online-only supplementary material). Most participants were aged 30 to 40, had completed secondary school, were married and were non-white. MS was detected in approximately 14.2% of the sample, and the following distribution of cardiovascular risk according to FRS was found: low 95.0%, moderate 4.1% and high 0.9% (Table 1).

**Figure 1:**
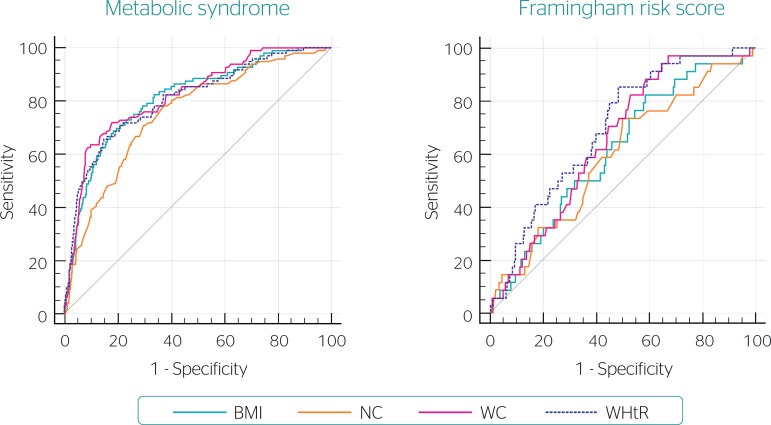
Receiver operating characteristic curves of the sensitivity and specificity of several anthropometric indicators to discriminate cardiovascular risk among rotating shift workers and two risk models: metabolic syndrome and the Framingham risk score. Inconfidentes, Brazil, 2011 (n = 678) BMI: body mass index; NC: neck circumference; WC: waist circumference; WHtR: waist-to-height ratio.

Figure 1 depicts the ROC curves for the anthropometric indicators and risk models. The AUC was >0.50 in all cases, which indicates satisfactory discriminatory power to predict CV outcomes (Figure 1, Table 2). For MS, the Hanley and McNeil test results indicated that BMI, WC and WHtR were statistically similar and superior to NC. For FRS, the AUC for WHtR was significantly larger than all other indicators. Cut-off points were calculated based on the highest Youden’s index (Table 2).

**Table 2:** Cut-off points for and performance of body fat indicators as predictors of cardiovascular risk among rotating shift workers in two risk models: metabolic syndrome and the Framingham risk score. Inconfidentes, Brazil, 2011 (n = 678)

Metabolic syndrome (NCEP-ATP III)					Framingham risk score			
	CP	AUC	95%CI	Youden's index	CP	AUC	95%CI	Youden's index
BMI kg/m^2^	≥29.1	0.81*	0.78-0.84	0.50	≥25.5	0.62*^,‡^	0.58-0.65	0.24
WC cm	≥97.7	0.82*	0.79-0.85	0.54	≥86.7	0.66^†^	0.62-0.69	0.30
WHtR	≥0.57	0.81*	0.78-0.85	0.51	≥0.53	0.70*	0.66-0.73	0.37
NC cm	≥40.4	**0.75** ^†^	0.72-0.78	0.41	≥39.2	0.60^†,‡^	0.56-0.63	0.24

AUC: area under curve; 95%CI: 95% confidence interval; CP: cut-off point; BMI: body mass index; NCEP-ATP III: National Cholesterol Education Program Adult Treat- ment Panel III; NC: neck circumference; WC: waist circumference; WHtR: waist-to-height ratio. AUC values greater than 0.70 are indicated in bold. * ^† ‡^ Hanley and McNeil test results are presented as symbols; there was no significant difference in AUC for body fat indicators marked with the same symbols (p>0.05).

Sensitivity, specificity, and the positive and negative predictive value of the selected body fat indicators (alone and in combination) are described in Tables 3 and 4. The sensitivity of WHtR and BMI for discriminating CV risk was more than 70% in both risk models; the corresponding cut-off points were predictive of CV risk.

**Table 3:** Sensitivity, specificity, and positive and negative predictive value of body fat indicators, alone and in combination, to predict cardiovascular risk according to the metabolic syndrome model among rotating shift workers. Inconfidentes, Brazil, 2011 (n = 678)*

Indicators	Metabolic syndrome			
	Sen	Spc	PPV	NPV
BMI	89.6 (81.9-94.2)	39.9 (36.0-43.9)	19.7 (16.3-23.7)	95.9 (92.6-97.7)
WC	60.4 (50.4-69.6)	92.1 (89.6-94.0)	55.8 (46.2-64.9)	93.4 (91.0-95.1)
WHtR	93.8 (87.0-97.1)	32.3 (28.6-36.2)	18.6 (15.4-22.3)	96.9 (93.4-98.6)
NC	81.3 (72.3-87.8)	57.3 (53.3-61.3)	23.9 (19.6-28.8)	94.9 (92.0-96.7)
BMI and WC	61.5 (51.5-70.6)	91.8 (89.2-93.7)	55.1 (45.7-64.2)	93.5(91.2-95.3)
BMI and WHtR	88.5 (80.6-93.5)	43.5 (39.5-47.5)	20.5 (16.9-24.7)	95.8 (92.7-97.7)
BMI and NC	81.3 (72.3-87.8)	62.5 (58.5-66.3)	26.4 (21.7-31.7)	95.3 (92.7-97.0)
WC and WHtR	61.5 (51.5-70.6)	91.8 (89.2-93.7)	55.1 (45.7-64.2)	93.5 (91.2-95.3)
WC and NC	87.5 (48.3-67.7)	92.3 (89.8-94.2)	55.5 (45.7-64.8)	93.1 (90.7-94.9)
NC and WHtR	81.3 (72.3-87.8)	61.3 (57.3-65.2)	25.7 (21.1-31.0)	95.2 (92.5-96.9)

BMI: body mass index (kg/m^2^); NC: neck circumference; NPV: negative predictive value; PPV: positive predictive value; Sen: sensitivity; Spc: specificity; WC: waist circumference (cm); WHtR: waist-to-height ratio.

* Cut-off points: BMI e 25 kg/m2; WC e 102 cm; WHtR e 0.5; NCe39.5 cm.

**Table 4:** Sensitivity, specificity, positive and negative predictive value of body fat indicators, alone and in combination, to predict cardiovascular risk according to the Framingham risk score among rotating shift workers. Inconfidentes, Brazil, 2011 (n = 678)*

Indicators	Framingham risk score			
	Sen	Spc	PPV	NPV
BMI	82.4 (66.5-91.7)	36.7 (33.0-40.4)	6.4 (4.5-9.1)	97.5 (94.7-98.9)
WC	23.5 (12.4-40.0)	85.1 (82.1-87.6)	7.7 (3.9-14.5)	95.5 (93.5-96.9)
WHtR	94.1 (80.9-98.4)	29.8 (26.4-33.5)	6.6 (4.7-9.2)	99.0 (96.3-99.7)
NC	61.8 (45.0-76.1)	52.6 (48.7-56.4)	6.4 (4.3-9.6)	96.3 (93.8-97.8)
BMI and WC	26.5 (14.6-43.1)	84.8 (81.8-87.4)	8.4 (4.5-15.2)	95.6 (93.6-97.0)
BMI and WHtR	82.4 (66.5-91.7)	40.1 (36.4-43.9)	6.8 (4.7-9.6)	97.7 (95.1-99.0)
BMI and NC	58.8 (42.2-73.6)	57.1 (53.3-60.9)	6.8 (4.4-10.2)	96.3 (93.9-97.8)
WC and WHtR	26.5 (14.6-43.1)	84.8 (81.8-87.4)	8.4 (4.5-15.2)	95.6 (93.6-97.0)
WC and NC	26.5 (14.6-43.1)	85.7 (82.8-88.2)	8.9 (4.8-16.1)	95.7 (93.7-97.1)
NC and WHtR	61.8 (45.0-76.1)	56.2 (52.3-60.0)	6.9 (4.6-10.4)	96.5 (94.2-98.0)

BMI: body mass index (kg/m2); MS: metabolic syndrome; NC: neck circumference; NPV: negative predictive value; PPV: positive predictive value; Sen: sensitivity; Spc: specificity; WC: waist circumference (cm); WHtR: waist-to-height ratio.

* Cut-off points: BMI e 25 kg/m2; WC e 102 cm; WHtR e 0.5; NC e 39.5 cm.

## DISCUSSION

The body fat indicators had adequate discriminatory power in both risk models. BMI, WC and WHtR were similar for MS, while WHtR had the highest discriminatory power for FRS. The discriminant power of these indicators for CV risk was further corroborated by the obtained sensitivity estimates, since the higher the sensitivity, the higher the efficacy of a test or indicator.^25^ Sensitivity was highest for WHtR in both the MS and FRS models.

In combined analysis, the WHtR + BMI (≥ 25 kg/m^2^) combination had the highest sensitivity in both risk models. Several authors recommend combining variables (i.e. indicators) to increase the sensitivity and specificity of diagnostic tests.^25^ Either alone or in combination, WHtR had the highest efficiency and sensitivity to predict CV risk in both risk models. BMI and NC, either alone or in combination, could discriminate risk in the MS model, and BMI could discriminate risk the FRS model.

A combination of BMI, WC and WHtR did not improve their ability to predict high CV risk.^26^ In another study performed in Brazil, WC and WHtR predicted global CV and high coronary risk better than BMI.^4^ In our study, BMI, WC and WHtR had similar discriminatory power for MS. While BMI is traditionally used in clinical practice and epidemiological studies, we suggest adding other body fat indicators when screening for CV risk.

Although many studies have compared the power of body fat indicators to discriminate CV risk in different models, few have included NC. The advantages of NC are that it is not influenced by postprandial abdominal distension and that it does not require disrobing. Studies in Brazil and Puerto Rico found that NC has satisfactory predictive power regarding cardiometabolic risk factors.^5^^,^^6^ However, in the present study NC had significant predictive power in both the FRS and MS models, and several authors have suggested combining it with WC to diagnose MS^5^. Stable et al.^5^ found a significant correlation between NC and abdominal fat. They also described an innovative approach to investigating body fat distribution patterns that included NC among the risk factors for MS. Joshipura et al.^6^ studied overweight people in Puerto Rico and found that the rate of MS was higher among those with high WC and NC.

In addition to analyzing the predictive power of body fat indicators, it is necessary to establish cut-off points as a basis for public health recommendations and epidemiological studies. The World Health Organization recommends setting specific cut-off points for different populations.^27^ In the present study we calculated representative cut-off points for rotating shift workers in the metropolitan region of Inconfidentes, Minas Gerais, Brazil according to two CV risk models. The cut-off points for WHtR, 0.52-0.53, were similar to those obtained in a study on adults in Salvador, Bahia, Brazil.^4^ A similar cut-off point (0.50) was reported in a South African study to identify the other two components of MS.^4^ These findings indicate that WHtR does not vary much in different populations and CV risk models. Moreover, in the present study WHtR had the best sensitivity/specificity relationship and the highest positive and negative predictive value.

Regarding WC, a Brazilian study by Barbosa et al.^28^ found that a cut-off point close to 90 cm efficiently detected high coronary risk and MS components. However, in a multicenter Latin American study, a cutoff point 91 cm best discriminated high coronary risk.^29^ The lack of consensus notwithstanding, the cut-off points obtained for Brazilian and larger Latin American populations were relatively close to ours.

The BMI cut-off points we obtained are similar to those recommended by the World Health Organization for white populations.^9^ In a study performed in six Latin American countries, the cut-off point for high coronary risk was 26 kg/m^2^.^30^

The cut-off point for NC was 39-40 cm, which agrees with that obtained by Ozkaya et al.^31^ to predict MS among a Turkish population. This range is similar to what has been recommended in the literature for obstructive sleep apnea, which is also associated with body fat and CV risk.^32^ Although few studies have sought to determine cut-off points for NC, the values we obtained are similar to those reported in the literature. Despite poorer discriminatory power for both MS and FRS, NC has been recommended for clinical practice, since this measurement is quick and easy and does not vary throughout the day.^33^

Since our sample consisted exclusively of male shift workers, the results cannot be generalized to other populations. Prospective studies are needed to analyze the validity of these cut-off points and establish causal relationships between anthropometric indicators and CV risk factors. Anthropometric data can be used in clinical practice and epidemiological studies, since they enable low-cost, easy-to-take measurements of body fat patterns that are associated with cardiometabolic risk.^2^ Some authors consider WHtR to be the best body fat indicator for predicting CV risk, given that there is an acceptable amount of fat in the upper part of the body according to height. Adjusting for height enables the definition a single cut-off point applicable to the general population independently of sex, age or ethnicity.^3^^,^^4^^,^^21^

## CONCLUSION

In this sample of shift workers, WHtR was the best body fat indicator for predicting CV risk in both the FRS and MS risk models. In addition, it is an easy and low-cost measurement with high clinical applicability for CV risk screening. Thus, the results of the present study could help with health risk detection due to poor body composition.
